# Assessing Cognitive Abilities of Patients With Shift Work Disorder: Insights From RBANS and Granger Causality Connections Among Resting-State Networks

**DOI:** 10.3389/fpsyt.2020.00780

**Published:** 2020-08-06

**Authors:** Yanzhe Ning, Kuangshi Li, Yong Zhang, Pei Chen, Dongqing Yin, Hong Zhu, Hongxiao Jia

**Affiliations:** ^1^ The National Clinical Research Center for Mental Disorders & Beijing Key Laboratory of Mental Disorders, Beijing Anding Hospital, Capital Medical University, Beijing, China; ^2^ Department of Rehabilitation, Dongzhimen Hospital, The First Affiliated Hospital of Beijing University of Chinese Medicine, Beijing, China

**Keywords:** granger causality connection, resting-state functional magnetic resonance imaging, resting-state networks, shift work disorder, cognitive abilities

## Abstract

**Background:**

Numerous studies have confirmed that long-term shift work is not only associated with increased health problems and acute impact on safety but also with impaired cognitive abilities. However, very little is known about effects of shift work on cognition-related brain resting-state networks. The aim of this study was to explore the effects of shift work disorder (SWD) on granger causality connection among resting-state brain networks.

**Methods:**

Thirty patients with SWD and 25 matched healthy subjects were recruited to undergo the Repeatable Battery for the Assessment of Neuropsychological Status (RBANS) and resting-state fMRI scanning. We employed independent component analysis (ICA) to extract resting-state brain networks and granger causality analysis (GCA) to characterize the difference of granger causality connection among cognition-related resting-state brain networks.

**Results:**

Compared with healthy subjects, patients with SWD showed impairments on the attention and immediate memory. Seven resting-state brain networks were identified, and patients with SWD showed more numerous granger causality connections in comparison with healthy subjects. Two-sample *t* test results showed that there were significantly increased inflows from the anterior default mode network (aDMN) to sensorimotor network (SMN) and left frontoparietal network (LFPN) to salience network (SN). Correlation analyses showed that the increased inflows from aDMN to SMN were negatively associated with the score of attention, while LFPN to SN were negatively associated with the score of visuospatial/constructional ability.

**Conclusions:**

This study indicates that SWD impairs cognitive performance, and the specific intrinsic brain granger causality connectivity among resting-state networks in SWD patients is affected after long-term shift works.

## Introduction

Shift work disorder (SWD) is a condition deﬁned by excessive sleepiness or insomnia accompanied by total sleep time reduction (1). According to the epidemiology, SWD effects approximately 10–38% of the shift worker population. Nurses make up the largest proportion of shift workers (15–20%) (2). Accumulating evidence suggests that long-term shift work not only affects work efficiency and satisfaction but also leads to physical and mental health problems (3, 4). In hence, given enough time, SWD may lead to more severe disorders, such as cardiovascular diseases, cerebrovascular events, metabolic disorders, gastrointestinal complaints, and multiple forms of cancer (5–7). Meanwhile, SWD also impairs memory, which will directly affect work efficiency or even cause safety consequences not only for the individuals concerned but also for society (8). Therefore, it is essential to evaluate impairments of cognitive abilities for patients with SWD comprehensively and uncover related neuroimaging mechanisms.

In the past decades, functional magnetic resonance imaging (fMRI) develops rapidly, which supplies a noninvasive and convenient method to explore the neuroimaging mechanisms of cognition declines. Numerous fMRI studies have revealed great changes of the brain’s intrinsic functional connectivity stemming from cognitive impairments on patients with sleep disorder (9–11). During resting state, brain functional networks including anterior default mode network (aDMN), posterior default mode network (pDMN), sensorimotor network (SMN), left frontoparietal network (LFPN), right frontoparietal network (RFPN), executive control network (ECN), visual network (VN), auditory network (AN), salience network (SN), cerebellum network, and language network are detected by fMRI, which can reflect spontaneous fluctuations and are associated with processing of cognition, emotion, action, and so on (12, 13). Patients with primary insomnia showed increased global functional connectivity strength in the ECN, aDMN, dorsal attention network (DAN), and VN (11). Previous studies have confirmed that DMN connectivity altered during sleep deprivation (14, 15). As another type of sleep disorder, to our best of knowledge, there was only one neuroimaging study, which reported that patients with SWD showed brain perfusion changes in multiple brain areas significantly correlated with insomnia severity (16).

In the study of causal relationships among brain networks, the causality model is suitable to display intranetwork communications, especially information flows among resting state network. The granger causality analysis (GCA) is always applied in studying causalities among brain regions or networks (17). It has been widely used in researches on stroke (18), Alzheimer’s disease (19), and so on. However, to our knowledge, there is no study focused on causal relationships of cognition-related brain networks in patients with SWD.

In the current study, we firstly recruited female nurses with SWD as research subjects and evaluated impairments on cognitive abilities. Then, all subjects underwent resting-state fMRI scanning. We extracted resting-state brain networks by independent component analysis (ICA) and applied the multivariate granger model to analyze the intranetwork causality in all subjects. We postulated that there were two different causal connectivity pattern and significant alterations in the seven important cognition-related brain networks in patients with SWD compared with healthy subjects.

## Materials And Methods

This study was approved by the Beijing Anding Hospital of Ethics Committee. All participants signed informed consents before inclusion in this study.

### Participants

Thirty right-handed participants (all females, aged 28.33 ± 2.60 years) met the following inclusion criteria: diagnosed as SWD according to the International Classification of Sleep Disorders (2nd edition) by the American Sleep Disorders Association (20); female nurses working at Beijing Anding Hospital; aged from 18 to 40 years, right-handed; continuous regular night shift for at least 1 year and work for 5 to10 years, at least two shift work per week; with no history of prophylactic or therapeutic medicine in the past 3 months; and with no history of long-term use of analgesics. The exclusion criteria were as follows: pregnant or lactation, history of neurologic or psychiatric disorders, participating in such cognitive experiments within the previous 1 year, any other health disorders or poor physical conditions that may influence participation, any brain structure damage or abnormalites identified by MRI examinations, history of alcohol or any drug dependency, and any MRI contraindications. Another 25 healthy subjects (all females, aged 27.19 ± 2.47 years) were recruited. They followed the inclusion criteria below: relative regularity of sleep in the past 12 months; aged 18 to 40 years, right-handed; and sleeping less than three times per month after 23 o’clock in the latest 1 year and night shift less than three times a month in the previous 1 year.

### Cognition and Symptoms Assessment

Prior to MRI scanning, each participant was asked to complete the Repeatable Battery for the Assessment of Neuropsychological Status (RBANS) (21) and Pittsburgh Sleep Quality Index (PSQI) to evaluate her attention and sleep quality (22).

RBANS, a cognitive screening test including 12 subtests, was utilized as the measure of global cognition. The RBANS generates five domain-specific index scores applied to evaluate five cognitive abilities: immediate and delayed memory, language, visuospatial/constructional ability, and attention. In spite of delayed memory, a component is based on four subtests, the other four components are based on two subtests. The immediate memory index comprises the story memory and list learning subtests, the visuospatial/constructional index comprises the line orientation and figure copy subtests, and the language index comprises the photo naming and semantic fluency subtests. The attention index comprises the digit span and coding subtests, and the delayed memory index comprises the list recall, story recall, list recognition, and figure recall subtests. The Chinese version of the RBANS translated by Cheng et al. was adopted in the current study (23). The test undergoes about 30 min. A trained neuropsychologist performed the test according to standardized procedures.

### MRI Acquisition

Images were acquired applying a 3.0 Tesla MRI scanner (Siemens, Prisma, Germany) at Anding Hospital, Beijing, China. Prior to scanning, all participants were asked to rest for 30 min before scanning. They were instructed to stay still, think of nothing in particular, keep eyes closed, and not to fall asleep during scanning. Earplugs were worn to attenuate scanner noise. The foam head holders were immobilized to minimize head movements during scanning.

Prior to the functional scanning, we collected high-resolution structural information for anatomical localization by using 3D MRI sequences. The resting-state fMRI data were collected using a single-shot, gradient-recalled echo-planar imaging sequence with the following parameters: repetition time = 2000 ms, echo time = 30 ms, flip angle = 90°, matrix = 64 × 64, field of view = 225 mm × 225 mm, slice thickness = 3.5 mm, gap = 1 mm, 32 interleaved axial slices, and 180 volumes.

In this study, a 490-s resting were scanned first, and then 250-s high-resolution structural scan were employed.

### Data Processing

Results included in this manuscript come from preprocessing performed using fMRI Prep 1.5.0 (24), which is based on Nipype 1.2.2 (25).

#### Anatomical Data Preprocessing

N4BiasFieldCorrection (26) [antsApplyTransforms (ANTs) 2.2.0] was applied for intensity non-uniformity (INU) to correct the T1 images. OASIS30ANTs as a target template was then used to skull-strip the T1 images. Cerebrospinal fluid, white matter, and gray matter were extracted by the command ‘fast’ (FSL5.0.9). Brain surfaces were reconstructed using recon-all (FreeSurfer 6.0.1). During reconstruction, the brain mask was estimated by the method to re-reconcile ANT-derived and FreeSurfer-derived segmentations (27). Brain structure abnormalites were identified by MRI examinations on two subjects. In hence, the two subjects were ruled out during anatomical data preprocessing.

#### Functional Data Preprocessing

First, a custom methodology of fMRIPrep was applied to generate a reference volume and its skull-stripped version. The BOLD reference was co-registered to the T1w reference by bbregister (FreeSurfer). Head-motion parameters of BOLD reference were estimated by mcflirt (FSL 5.0.9). After that, slice time was performed using 3dTshift (AFNI). The processed time series were resampled to surfaces (fsaverage5). Framewise displacement (FD), DVARS, and three region-wise global signals were calculated for preprocessed BOLD. Some physiological regressors were also extracted for noise correction (28). Four participants were ruled out for exhibiting head motion of >1.5° rotation maximum translation and 1.5 mm in the process of MRI scanning. Finally, voxel-based resampling was implemented by ANTs. Surface resampling was implemented by mri_vol2surf (FreeSurfer).

#### RSNs Extraction

ICA was applied to extract the RSNs by GIFT software (University of New Mexico, Albuquerque, NM). The number of independent components in all data was calculated by method of the minimum description length (MDL) technique. Thirty components were estimated. Randlnit and Bootstrap operations were applied to evaluate the independent components. According to research objectives, seven independent components were selected based on the largest spatial correlation comparing with previous resting brain network templates. We selected seven specific networks including ECN, VN, sensory motor network (SMN), LFPN, pDMN, SN, and aDMN.

#### Network Granger Analysis

All selected components were filtered between 0.01 and 0.1Hz for multivariate granger causal modal to explore the characteristics of networks. Meanwhile the generalized partial directed coherence (GPDC) was selected as the measured parameter (29). The order of GCA was determined by the method of Akaike information criterion. Then, comparisons between groups were analyzed on causal interaction of seven components. One-sample t test in each component was also applied to compute the single network imaging. For comparing the patient group with control group, P value of two sample t test was set as 0.05, which was corrected by false discovery rate (FDR) for multiple comparisons. Finally, BrainNet Viewer was used to display the result onto a 3D brain surface.

### Correlation Analysis

As we conducted a comparison between patients with SWD and healthy subjects, we found the patients showed increased inflows from the aDMN to SMN, LFPN to SN. Mean granger causality values between the aDMN and SMN, or LFPN and SN, correlated with the scores of REBANS using Pearson correlation analysis. Statistical analyses were conducted using SPSS 20.0 (SPSS Inc., Chicago, IL, USA), and threshold was set at *P* < 0.05.

## Results

### Demographic and Clinical Information

Socio-demographic characteristics and PSQI scores of all subjects are displayed in **Table 1**. **Table 1** also showed that the age of patients with SWD distributed between 25 and 30 years old, and all subjects are female, which can eliminate ageing and gender impacts on changes in tolerance to shiftwork.

**Table 1:** The demographic information of patients with SWD and healthy controls.

Items	Patients with SWD (N = 30)	Healthy controls (N = 25)
Gender (male/female)	0/30	0/26
Age (years)	28.33 ± 2.60^*^	27.19 ± 2.47
Educational level (years)	17.35 ± 1.06^#^	17.14 ± 0.64
PSQI scores	9.60 ± 3.83	/

^*^Results from two-sample independent t test of the comparison between two groups, t = 1.68, P = 0.10 (for age); ^#^results from two-sample non-parametric test of the comparison between two groups, z = -0.18, P = 0.86 (for educational level).

Compared with healthy subjects, patients with SWD showed declines on attention (t = -9.62, *p* < 0.0001) and immediate memory (t = -5.10, *p* < 0.0001). No significant difference on visuospatial/constructional (t = -0.84, p = 0.40), language (t = -1.89, p = 0.06), and delayed memory (t = -1.20, p = 0.24) was found between the two groups (shown in **Table 2**).

**Table 2:** The results of RBANS between patients with SWD and healthy controls.

RBANS index score	Patients with SWD (N = 30)	Healthy controls (N = 25)
Immediate memory	90.33 ± 5.88*	99.16 ± 6.96
Visuospatial/Constructional	103.50 ± 4.67	104.56 ± 4.60
Language	96.57 ± 5.76	99.76 ± 6.76
Attention	85.27 ± 7.69*	103.40 ± 5.96
Delayed memory	96.90 ± 4.26	98.36 ± 4.78

Results from two-sample independent t-test of the comparison between two groups, *p＜0.05. RBANS: Repeatable Battery for the Assessment of Neuropsychological Status.

### ICA Results

**Figure 1:**
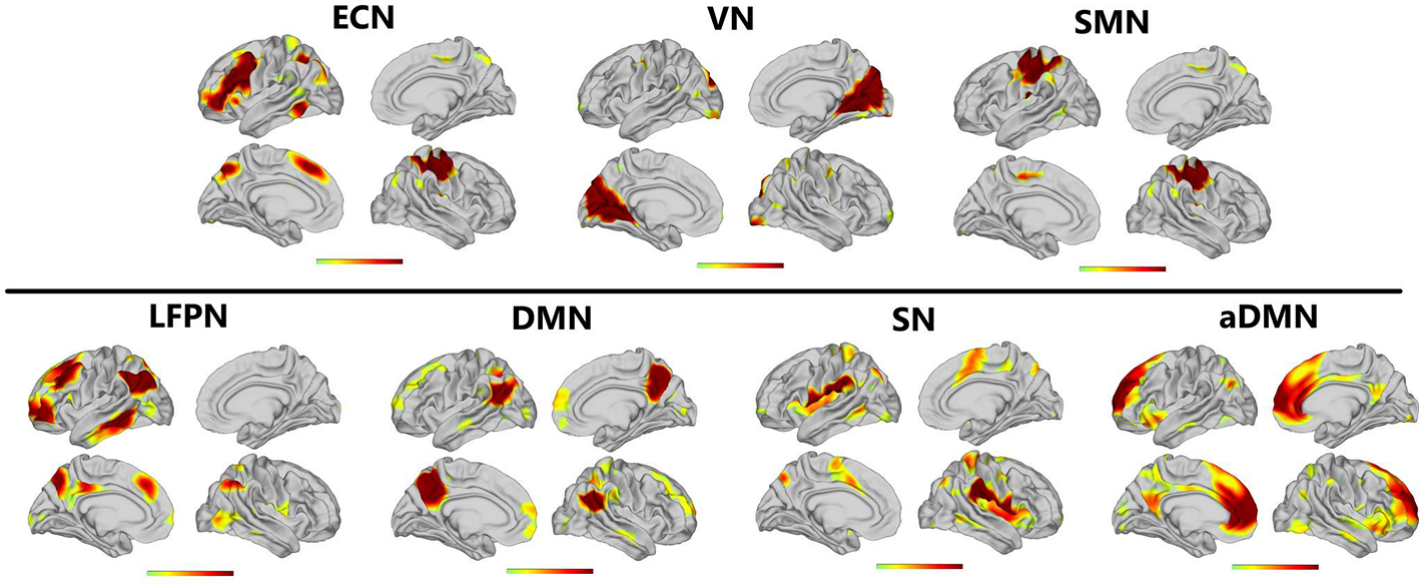
Resting-state networks screened through ICA. The t value (depicted by cold to warm colors) represents the spatial statistical signiﬁcance of the current networks. L, left; R, right.

Applying ICA in all participants, the SN, aDMN, pDMN, ECN, LFPN, SMN, and VN were extracted. Spatial positional distributions of the seven resting-state networks are shown in **Figure 1** and **Table 3**.

**Table 3:** Spatial positional distributions of brain networks.

**Region**	**Hem**	**MNI coordinates**			**t value**	**Area(mm)**
		X	Y	Z		
**Executive control network**						
Inferior frontal cortex	L	41	28	24	12.16	4199
Intra parietal cortex	L	33	65	42	17.38	2003
Anterior cingulate	L	7	26	47	6.45	550
Temporal cortex	L	54	51	8	6.22	612
Insular	L	28	22	1	4.23	95
Parieto-occipital cortex	L	5	71	45	7.01	432
Prefrontal cortex	R	-46	-25	-28	10.67	2081
Intraparietal cortex	R	-35	-66	-43	10.55	611
Anterior cingulate	R	-7	-40	-40	5.57	460
Insular	R	-30	-22	-1	4.49	156
**Visual network**						
Posterior cingulate	L	-13	-75	12	20.60	9604
Posterior cingulate	R	20	-66	7	19.68	6824
**Sensory motor network**						
Precentral cortex	L	-44	-25	61	28.94	4552
Postcental cortex	R	45	-22	60	23.15	4102
**Left frontoparietal network**						
Dorsolateral prefrontal cortex	L	-41	-28	-24	12.16	5400
Superior parietal cortex	L	-33	-65	-42	17.37	2427
Posterior cingulate	L	-5	-71	-45	7.01	679
Posterior temporal cortex	L	-54	-51	-8	6.22	777
Medial prerontal cortex	L	-7	-26	-47	6.45	776
Superior parietal cortex	R	50	-55	-43	5.61	611
Medial superior temporal cortex	R	46	-77	0	3.14	676
**Posterior default mode network**						
Superior parietal cortex	L	-52	-54	23	12.89	3162
Posterior cingulate cortex	L	-8	-61	44	16.01	1935
Superior parietal cortex	R	51	-49	25	13.75	1668
Posterior cingulate	R	5	-61	38	16.5326	1464
Superiror temporal cortex	R	60	-16	-15	3.2	771
**Salience network**						
Insular	L	-62	-30	21	10.83	5207
Middle cingulate	L	-10	7	40	3.29	867
Postcentral cortex	L	-27	-32	68	3.43	977
Superior parietal cortex	L	-32	-73	43	4.53	527
Insular	R	-60	-29	20	10.8	6237
Postcentral cortex	R	24	-46	62	4.4	1312
Middle cingulate	R	7	3	54	3.49	1235
**Anterior default mode network**						
Anterior cingulate	L	-7	60	18	9.5	9306
Anterior cingulate	R	8	58	13	8.68	9974
Medial superior temporal cortex	R	45	-66	-12	2.2	537

### GCA Results

**Figure 2:**
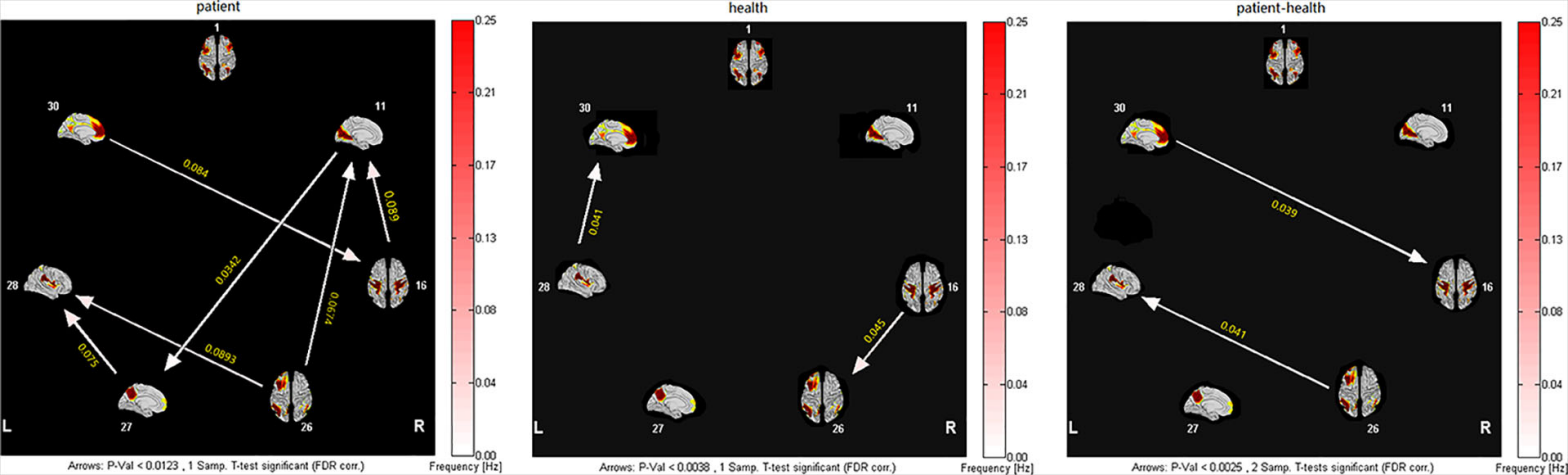
Inter- and intra-group comparisons of patients with SWD and healthy subjects. Panels represent visual descriptions of causal connectivity between 2 networks among the 7 resting-state networks, including 1, ECN; 11, VN; 16, SMN; 26, LFPN; 27, pDMN; 28, SN; 30, aDMN. Arrow directions represent cause and effect. Values on the color bar (corresponding with arrow colors) demonstrate frequency at which causality was found. Left panel: One-sample t test result of inter-group intranetwork causal relationship of MwoA patients. Center panel: One-sample t test result of intergroup intranetwork causal relationship of healthy subjects. Right panel: Two-sample t test result of intra-group intranetwork causal relationship of MwoA patients minus healthy subjects.

The granger causality of the seven resting-state brain networks in patients with SWD showed different patterns of causal connections compared with healthy subjects. Patients showed more numerous granger causality connections. For the patients, SN and VN were the core networks with more effective connections than others (**Figure 2**, left panels). Two-sample *t* test results showed that there were significantly increased inflows from aDMN to SMN and LFPN to SN (shown in **Figure 2**).

### Correlations

**Figure 3:**
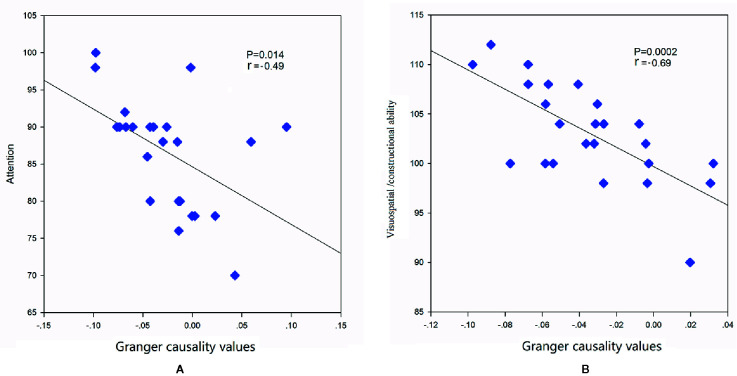
Correlations between mean granger causality values within abnormal casual connectivity and the scores of REBANS. **(A)** The mean granger causality values from aDMN to SMN were negatively associated with the score of attention (r = -0.49, p = 0.014). **(B)** The mean granger causality values from LFPN to SN were negatively associated with the score of visuospatial/constructional ability (r = -0.69, p = 0.0002).

To investigate the association between cognitive performances and abnormal casual connectivity, we conducted correlation analysis between mean granger causality values within abnormal casual connectivity and the scores of REBANS. As shown in **Figure 3**, the correlation analysis revealed that the increased inflows from aDMN to SMN were negatively associated with the score of attention (r = -0.49, p = 0.014), while LFPN to SN were negatively associated with the score of visuospatial/constructional ability (r = -0.69, *p* = 0.0002).

## Discussion

In the present study, we attempted to assess cognitive abilities and granger causality connection among cognition-related brain networks on patients with SWD. Our results revealed that patients with SWD showed declines on attention and immediate memory, more numerous granger causality connections among resting-state brain networks than healthy subjects, significant causal relations from aDMN to SMN and LFPN to SN were observed in comparison with healthy subjects. Moreover, the increased inflows were negatively associated with the score of attention and visuospatial/constructional ability.

Shift work disrupts the body’s circadian rhythms. Circadian disruption is reliably associated with mood disorders, cognitive function and subjective wellbeing (30). Displaced work hours could change lifestyle factors for shift workers, which may impair cognitive functions (31). In this study, cognitive abilities were assessed using RBANS. Our results revealed that patients with SWD showed declines on attention and immediate memory. This ﬁnding is in line with previous studies. One recent research on shift workers showed that driving events were increased following night shifts, and inattention-related events were highest during the postnight shift commute compared with day and evening shifts, which pointed out that attention was impaired on SWD (32). Another study on sleep and alertness in SWD confirmed that the SWD group had more lapses in psychomotor vigilance tasks compared with the non-SWD group, which also revealed attention impairment in SWD (33). A large cross-sectional sample study showed that memory function tended to decrease with increasing shift-work duration for shift workers (34). An event-related brain potential study demonstrated significant attenuation of mismatch negativity amplitude over frontal regions in patients with SWD, which suggested sensory memory reduction in SWD (35). As circadian misalignment and sleep disruption are detrimental to hippocampus-dependent memory, shift work could impair memory and learning processes (36), which might interpret immediate memory impairment on patients with SWD.

Shift work can result in physiological stress, which have an impact on brain structures and function involved in cognition (37). In our study, resting-state brain functional networks were extracted from patients with SWD. We found that significant causal relations from aDMN to SMN and LFPN to SN were observed in comparison with healthy subjects. The aDMN, SMN, SN, and LFPN are mainly related to cognition and emotion processing. The aDMN is related to self-referential mental activity (38), while SN is the central region for detecting and filtering salient stimuli and modulates cognitive resources including attention for salient stimuli (39, 40). Both networks are associated with regulating emotion. Prior research indicated that SWD can lead to emotional dysregulation, such as negative mood states and frustration (41). Numerous studies revealed altered DMN connectivity during sleep deprivation and even normal variability in hours of sleep the night (14, 42). A system-neuroscience-based meta-analysis confirmed that attention deficit/hyperactivity disorder was associated with disrupted DMN (43). SMN involves in both somatosensory perception and movement generation (44). Circadian rhythmicity could modulate sensorimotor cortices (45), which suggested SWD could influence SMN. Our result also showed that increased inflows from aDMN to SMN were negatively associated with the attention, which may serve as a new potential biomarker for attention decline on SWD.

Patients with insomnia show less functional connectivity variability between the SN and the left executive-control network (ECN) (46). The SN is confirmed to modulate the activation and deactivation of ECN. Meanwhile, LFPN is considered to underpin executive control functions, memory and visual processing, and the ECN and LFPN both monitor executive control functions. A previous study on essential tremor showed that the increased connectivity of LFPN was associated with worse performance on visuospatial ability (47). Another study on obsessive compulsive disorder also revealed hypoconnectivity between frontoparietal control network (FPN) and SN (48). Hence, increased inflows from LFPN to SN in SWD, as well as its negative relationship with visuospatial/constructional ability, may indicate a compromised capability of patients with SWD to interact between the cognition and emotion. These findings interpreted that brain networks were widely hyperarousal even during daytime resting state in patients with SWD and might provide more details to understand the underlying neuromechanism of cognitive impairments and emotional dysregulation, including memory impairment and attention fatigue.

However, there are still several limitations in our current study. First, this is a cross-sectional study, and it is unclear how the cognitive impairments of SWD and disruptions in the granger causality connections change over time. Moreover, cognitive functions tend to decrease with the increases in the duration of exposure to shift work. Longitudinal studies are needed in the future. Secondly, as we only enrolled female participants in our study, it is unclear how granger causality connections and cognitive impairment change on male participants. It is necessary to study male patients with SWD in the future.

## Conclusion

This study indicates that SWD impair brain function and cognitive performance, and the specific intrinsic brain granger causality connectivity among resting-state networks in SWD patients are affected after long-term shift works.

## Data Availability Statement

The original data of this study are available from the corresponding authors upon reasonable request.

## Ethics Statement

The studies involving human participants were reviewed and approved by Beijing Anding Hospital of Ethics Committee. The patients/participants provided their written informed consent to participate in this study.

## Author Contributions

YN and HJ conceived and designed the study. YN and KL analyzed the data. YN, YZ, PC, DY, and HZ performed the experiment. YN, KL, and HJ drafted the manuscript and gave final approval of the manuscript.

## Conflict of Interest

The authors declare that the research was conducted in the absence of any commercial or financial relationships that could be construed as a potential conflict of interest.
